# Accumulation of Secretory Vesicles in the Lacrimal Gland Epithelia Is Related to Non-Sjögren's Type Dry Eye in Visual Display Terminal Users

**DOI:** 10.1371/journal.pone.0043688

**Published:** 2012-09-04

**Authors:** Mizuka Kamoi, Yoko Ogawa, Shigeru Nakamura, Murat Dogru, Toshihiro Nagai, Hiroto Obata, Masataka Ito, Minako Kaido, Tetsuya Kawakita, Yasunori Okada, Yutaka Kawakami, Shigeto Shimmura, Kazuo Tsubota

**Affiliations:** 1 Department of Ophthalmology, Keio University School of Medicine, Tokyo, Japan; 2 Department of Pathology, Keio University School of Medicine, Tokyo, Japan; 3 Department of Ophthalmology, Jichi Medical University, Tochigi, Japan; 4 Department of Developmental Anatomy and Regenerative Biology, National Defense Medical College, Saitama, Japan; 5 Division of Cellular Signaling, Institute for Advanced Medical Research, Keio University School of Medicine, Tokyo, Japan; Institute of Molecular and Cell Biology, Singapore

## Abstract

Previous observations in a rat model of a non-Sjögren's syndrome (non-SS) type of dry eye seen in users of visual display terminals (VDT) indicated that secretory vesicle (SV) accumulation in the lacrimal gland epithelia contributes to the condition. Here, to examine this possibility in humans, we compared the lacrimal gland histology and percent SV area in the cytoplasm of acinar epithelial cells using light microscopy and transmission electron microscopy, in patients with VDT work-related non-SS dry-eye (VDT group), SS-induced dry-eye, and autopsied normal controls. In addition, the VAMP8 (vesicle-associated membrane protein 8, an exocrine-pathway molecule) and Rab3D (mature vesicle marker) were histochemically examined in lacrimal gland tissue sections. The lacrimal gland acini were larger in the VDT group than in the SS group, and the percent SV area was significantly higher in the VDT group than in the normal controls (*P* = 0.021) or SS group (*P* = 0.004). Immunostaining revealed abnormal distributions of VAMP8 in the VDT and SS groups. Rab3D was more strongly expressed in the cytoplasm of acinar epithelial cells in the VDT group than in that of normal controls. The duration of VDT use was significantly longer in the VDT group than in the other groups. These findings suggest that excessive SV accumulation in the acinar epithelia may contribute to the reduced tear secretion in VDT users.

## Introduction

Work involving the use of VDTs (visual display terminals) has been increasing with the development of information technology all over the world. VDT use is prevalent both in the office environment and daily life. Various eye complaints such as burning, dryness, itching, eyestrain, and others are reported in VDT users [1]. Dry eye has been increasingly recognized as a cause of these symptoms in recent years. Although dry eye does not cause blindness, it is associated with eyestrain, redness, irritation, and decreased functional visual acuity that affect patients' quality of life (QOL) [2], [3]. Despite the fact that many computer users suffer from dry eye related to VDT work, the mechanism of VDT-related dry eye is uncertain. It has been proposed that a decreased blinking rate induces the excessive evaporation of tear fluid in VDT users [4]–[8]. In addition, we recently reported that there is a negative relationship between VDT use duration and tear secretion. We also showed that the lacrimal gland epithelia in a rat VDT-user model contained more secretory vesicles (SVs) than normal lacrimal gland epithelia [9]. This rat VDT-user model mimics human VDT users whose blinking rate is decreased by VDT work [10]. These findings suggested that VDT-related dry eye was not only caused by excessive evaporation, but also by lacrimal gland hypofunction in animal models [9].

Tearing is critical to the maintenance of the homeostasis of the ocular surface. Tear film consists of aqueous phase, mucins, and a lipid layer. Tears contain water, proteins, vitamins, and other materials [11]. Various eye drops and surgical treatments have been used to treat dry eye. However, the mechanism of dry eye has not been fully elucidated, and neither a radical treatment nor a prophylactic treatment has been established.

Although the fundamental mechanism is still uncertain, several mechanisms for dry eye have been proposed including tear hyperosmolarity and tear-film instability. Tear hyperosmolarity is induced by tear deficiency owing to lacrimal gland failure or increased evaporation of tear fluid. Tear film instability can be caused by dry eye, ocular allergy, preservative eye drop use, and contact lens wear [12]. These factors are closely related to each other and affect the cause of dry eye.

So far, two types of dry eye syndrome have been proposed: tear-deficient dry eye and evaporative dry eye. Tear-deficient dry eye is mainly caused by disorders of the lacrimal gland, and occurs in Sjögren's syndrome type and non-Sjögren's syndrome type (non-SS) dry eye. Evaporative dry eye is characterized by excessive evaporation of the tear film layer from the ocular surface, while tear secretion in normal [13]. It is caused by a decreased blinking rate, MGD (meibomian gland dysfunction), or contact lens wear.

However, we have proposed that a new type of dry eye, lacrimal gland hypofunction, occurs in a rat model of VDT use [9]. In our study, we have been interested in whether lacrimal gland hypofunction is involved in VDT work-related non-SS dry eye in humans. SS-type dry eye is often severe, and results from destruction of the lacrimal gland by lymphocytic infiltration. In contrast, in recent years we have treated several patients with non-SS dry eye who have decreased tear secretion. In these patients, large numbers of SVs appeared to accumulate in the epithelia of the lacrimal glands. These patients have characteristically worked long hours using a VDT for many years, and non-SS dry eye may be increasing among VDT users.

Based on our previous findings in rats, we hypothesized that VDT work-related non-SS dry eye in humans (VDT group) is partially induced by a failure of tear secretion, possibly because of an accumulation of SVs resulting from a decreased blinking rate, similar to animal models. So far, there is no study investigating the mechanism of dry eye involving SVs in human lacrimal gland. In this study, we focused on a human VDT group with mild ocular surface disorders and low tear secretion. The purpose of this study is to elucidate whether SV accumulation is involved in VDT work-related dry eye in human subjects.

## Materials and Methods

### Study Design

This is an exploratory study in humans, in which lacrimal gland samples from VDT users were examined histologically, to confirm the findings from a previously reported rat model that mimics the reduced blinking of VDT subjects. Lacrimal gland specimens were obtained by biopsy for the diagnosis of SS. Written informed consent was obtained from all patients. Normal lacrimal glands from autopsies were obtained by M.I. form National Defense Medical College and H.O. from Jichi Medical University. All the research and measurements followed the tenets of the Declaration of Helsinki. This study was also approved by the ethics committee of the Keio University School of Medicine, who also approved the use of autopsy samples. We obtained comprehensive written informed consent for the use of the autopsy samples, from the families of the deceased.

Lacrimal gland biopsy specimens were examined histologically using conventional techniques (hematoxylin and eosin [H&E] staining), transmission electron microscopy (TEM), and immunohistochemistry. The lacrimal gland biopsy specimens from VDT-related dry eye patients were compared with specimens from SS patients and autopsied normal controls (Table 1 A).

**Table 1:** Patient characteristics.

	VDT	SS	Normal
Number of cases	4	15	9
Gender (M/W)	2/2	0/15	7/2
Mean age	57.3	56.5	69.6
LE (Number of cases)	4	13	9
TEM (Number of cases)	4	12	4

VDT, VDT work related non-Sjögren's syndrome dry eye; SS, Sjögren's syndrome dry eye; M/W, man/woman; LE, light microscopy; TEM, transmission electron microscopy.

### Patients

We studied 4 patients with VDT work-related non-SS dry eye (2 males and 2 females, mean age; 57.3±11.1), 15 patients with SS (15 females, mean age; 56.5±10.2) who had diagnosed dry eye, and 9 normal controls (7 males and 2 females, mean age; 69.6±24.6) (Table 1, 2). SS was diagnosed when all the criteria of Fox et al were met [14], [15]: 1) keratoconjunctivitis sicca with Rose-Bengal or fluorescein staining on the ocular surface, 2) xerostomia, 3) minor salivary gland, lacrimal gland biopsy specimen including 1 focus/4 mm^2^, and 4) existence of rheumatoid factor or antinuclear antibody or antibodies to SSA or SSB.

**Table 2:** Clinical evaluation of ocular findings.

Case	LM	TEM	Age	Gender	F	RB	BUT	S	S (N)	VDT duration
VDT-1	○	○	43	M	6	4	4	0	0	14 h/day
*VDT-2*	○	○	65	F	0	0	3	1	(−)	6∼7 h/day
VDT-3	○	○	67	M	5	1	3	1	1	5∼6 h/day
*VDT-4*	○	○	54	F	0	2	3	2	16	10 h/day
SS-1	○	○	34	F	7	8	2	1	4	(−)
SS-2	○	○	68	F	6	6	5	0	(−)	1 h/day
SS-3	○	○	59	F	4	3	2	4	(−)	4 h/day
SS-4	○	○	68	F	6	7	2	0	2.5	1∼2 h/day
SS-5	×	○	53	F	9	7	(−)	1	2	1 h/day
SS-6	○	○	49	F	1	7	3	5	(−)	(−)
SS-7	○	×	59	F	6	5	(−)	1	16	(−)
SS-8	○	○	62	F	7	(−)	1	2	(−)	(−)
SS-9	○	×	54	F	2	(−)	3	2	(−)	2 h/day
SS-10	○	×	40	F	6	4	0	2	2	2 h/day
SS-11	○	○	65	F	1	0	4	1	5	(−)
SS-12	○	○	66	F	3	(−)	3	1	1	(−)
SS-13	○	○	66	F	9	9	(−)	5	4	(−)
SS-14	×	○	50	F	5	5	(−)	0	2	(−)
SS-15	○	○	54	F	9	8	0	1	1	unknown

VDT, VDT work related non-Sjögren's syndrome dry eye; SS, Sjögren's syndrome dry eye; LM, light microscopy; TEM, transmission electron microscopy; M, Male; F, Female; F, Fluorescein score; RB, Rose-Bengal score; BUT, Tear film break up time; S, Schirmer's test; S(N), Schirmer's test with nasal stimulation; VDT duration, Visual display terminals duration; h/day, hours per day; italics, These patients had been diagnosed as dry eye at the other institution and treated with eye drops.

SS dry eye was diagnosed when the tear film of patients showed a disturbance of tear dynamics (tear film break up time (BUT) ≤5 seconds, Schirmer's test ≤5 mm), ocular surface abnormalities (Rose-Bengal score ≥3, fluorescein score ≥1) and ocular symptoms in addition to Fox criteria [12], [16].

Non-SS dry eye was diagnosed when the Fox criteria were not met except for the dry eye criteria.

### Histology and Immunohistochemistry

Histological and immunohistochemical analyses were performed using formalin-fixed paraffin-embedded tissue sections.

For immunohistochemistry, specimens deparaffinized with xylene were soaked in ethanol for 20 minutes for hydrophilization. The sections were then immersed in 10% DAKO REAL™ Target Retrieval Solution (Dako Denmark) and heated in a microwave for 10 minutes. After cooling, the sections were blocked with 10% goat serum (Invitrogen, Carlsbad, USA) for 30 minutes and then incubated overnight at 4°C with the following primary antibodies: a rabbit monoclonal antibody against VAMP8 (vesicle-associated membrane protein 8) (1∶100 dilution) (Abcam, UK) or a rabbit polyclonal antibody against Rab3D (1∶50) (Protein Tech Group, Inc, Chicago, USA). After being washed with PBS, the sections were treated with a peroxidase-conjugated rabbit secondary antibody (Nichirei Corporation, Tokyo, Japan) for 45 minutes at room temperature, and then washed with PBS again. The reaction products were developed with a mixture of DAB Tris tablets (Muto Pure Chemicals Co., Ltd, Japan) and 0.3% H_2_O_2_. Nuclear staining was performed by treating with hematoxylin for 2 seconds. The sections were mounted and examined. Photographs were taken with a microscope (COOLSCOPE II, Nikon Corporation, Tokyo, Japan). All of the slides were reviewed twice by two independent observers (K.M. and Y.O.)

### Transmission electron microscopy (TEM)

A portion of lacrimal gland was fixed with 2.5% glutaraldehyde and subjected to examination by TEM as described previously [17]. One-micrometer-thick sections were stained with methylene blue and thin-sectioned were made with a diamond knife. The sections were collected on mesh grids, stained with uranyl acetate and lead citrate, and examined under an electron microscope (1230 EXII; JOEL, Tokyo, Japan). All photographs were taken with a bio scan camera (Gatan bio scan camera model 792, Tokyo, Japan).

### Analysis of histopathology and TEM images

**Figure 1:**
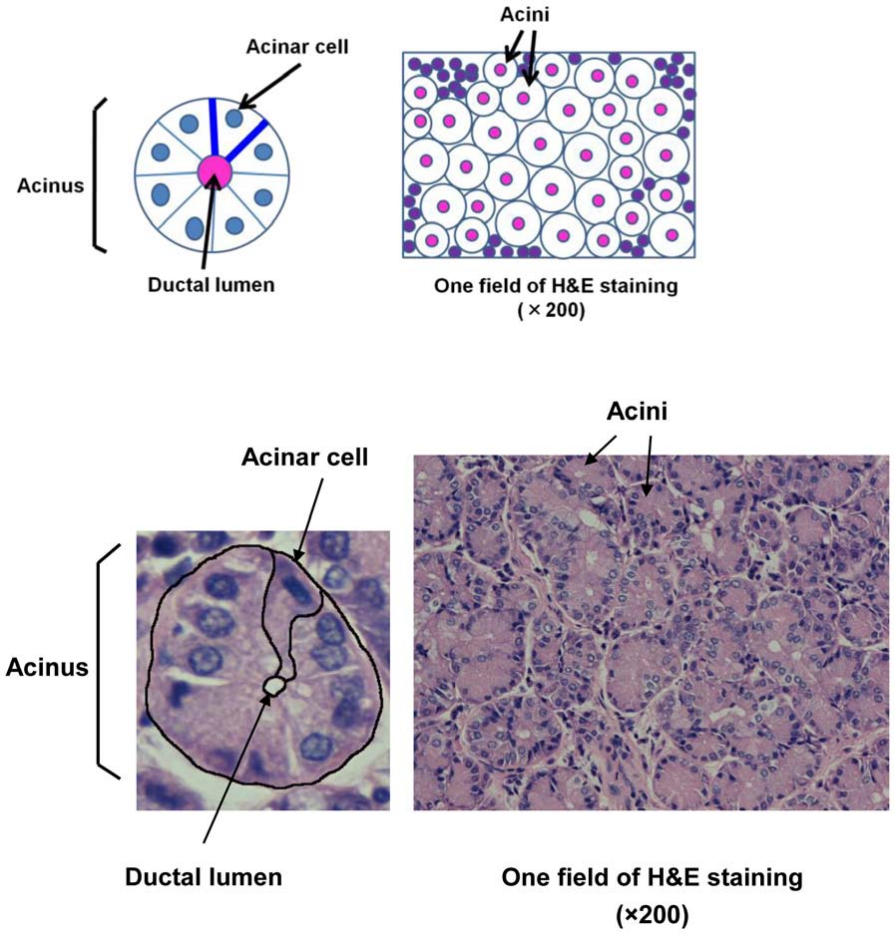
Scheme of the analytical method. (1) Percent area of the ductal lumen per acinus. (2) Percent area of the acini per field (×200). (3) Size of the lacrimal gland acinar cell.

All of the photographs were taken at random and assessed by two independent researchers in a blind manner using Photoshop CS4 (Adobe Systems Inc, Tokyo, Japan) and Scion Image software (Scion Corporation, Frederick, MD, USA; http://www.scioncorp.com) [18]–[20]. Scion Image is an open-source analysis software, available for free on the indicated web site. We analyzed samples from 4, 13, and 9 subjects for the VDT group, SS group, and normal controls, respectively. We determined the following values: (1) percent area of the ductal lumen per acinus, (2) percent area of the acini per field, and (3) the size of individual acinar cells relative to that of normal acinar cells (Figure 1). For the ductal lumen area/acinus, we calculated the ductal lumen area (in pixels) and the acinar area (in pixels) using Scion Image. The ductal lumen area was divided by the acinar area. For the acinar area/field, we calculated the acinar area (in pixels) and the area of one field (in pixels) using Scion Image. The acinar area was divided by the area of one field at ×200 magnification. For each sample, we used 3 representative photographs of H&E-stained sections (×200) for analysis. Since one photomicrograph of H&E-stained lacrimal gland tissue contains approximately 200 acini, the total number of analyzed acini was 879 (n = 4 cases), 1869 (n = 9 cases), and 2241 (n = 13 cases) in the VDT, normal controls, and SS groups, respectively. The mean number of acini analyzed per case was 219.75±29.66, 207.67±36.39, and 172.38±78.28 in the VDT group, normal controls, and SS group, respectively.

We performed TEM to examine the SVs in detail. For each sample, we analyzed 5 representative acini of the lacrimal gland by TEM (×2000). We used samples from 4, 4, and 12 subjects for the VDT group, normal controls, and SS group, respectively. The percent SV area of the cytoplasm of lacrimal gland epithelial cells was also determined for each group.

All the photographs were cropped to show a lacrimal gland acinus by Photoshop CS4. Images showing the ductal lumen area and SV area were generated using Photoshop CS4. These two types of images were then analyzed using the Scion Image software to obtain the acinar area, ductal lumen area, and SV area (all in pixels) in the lacrimal gland. We also counted the number of nuclei of the lacrimal gland acini. We used these data to calculate the above-mentioned values. In addition, we measured the perimeter of each SV with the Scion Image software and calculated the diameter from the circumference.

### Statistical Analysis

Statistical analysis was performed with the Mann-Whitney U test (SPSS19.0 for Windows, SPSS Japan Inc, Tokyo, Japan). Differences between measured variables were considered significant if the P value was <0.05. This study is an exploratory investigation using human lacrimal gland samples. Therefore, we used the Mann-Whitney U test for small sample sizes [21].

## Results

### Clinical ocular findings and analysis of the lacrimal gland histology by H&E staining

We examined the clinical ocular findings in the VDT and SS groups. There was no significant difference between these groups except for the Rose-Bengal score. The ocular findings of the SS group tended to be worse than those of the VDT group (Table 2). Both the stimulated and basal tearing were reduced in the VDT and SS groups.

We first confirmed that our samples were useful for comparing human VDT work-related non-SS dry eye, SS, and normal subjects. To do so, we first investigated the degree of lymphocytic infiltration by the Greenspan focus score. The results were 1.75±0.5, 1.67±0.5, and 2.70±1.38 in the VDT group, normal controls, and SS group, respectively. The SS group tended to have a higher score than the other 2 groups.

Next, we counted the number of infiltrated inflammatory cells in the H&E-stained samples. The total number of inflammatory cells was 130 (n = 4), 264 (n = 9), and 5172 (n = 13) in the VDT group, normal controls, and SS group, respectively. The mean number of inflammatory cells per sample was 10.8±14.8, 9.78±8.22, and 132.6±173.8 in the VDT group, normal controls, and SS group, respectively. There was a significant difference between the VDT group and SS group (*P* = 0.014), and between the normal controls and SS group (*P* = 0.004).

We also reexamined the X15000 TEM photographs for lipofuscin. Although there were some lipofuscin-like components in the TEM photographs of a normal subject at age 78 and a VDT patient at age 67, there were no significant differences among the three groups. These findings confirmed our diagnoses and verified that these samples were appropriate for analyzing the groups with the three clinical backgrounds examined in this study.

To examine the morphological characteristics of the lacrimal gland epithelia in the VDT group, the SS group, and normal controls, we first examined the percent area of the ductal lumen per acinus, the percent area of the acini per field, and the size of the individual acinar cells relative to normal acinar cells.

**Figure 2:**
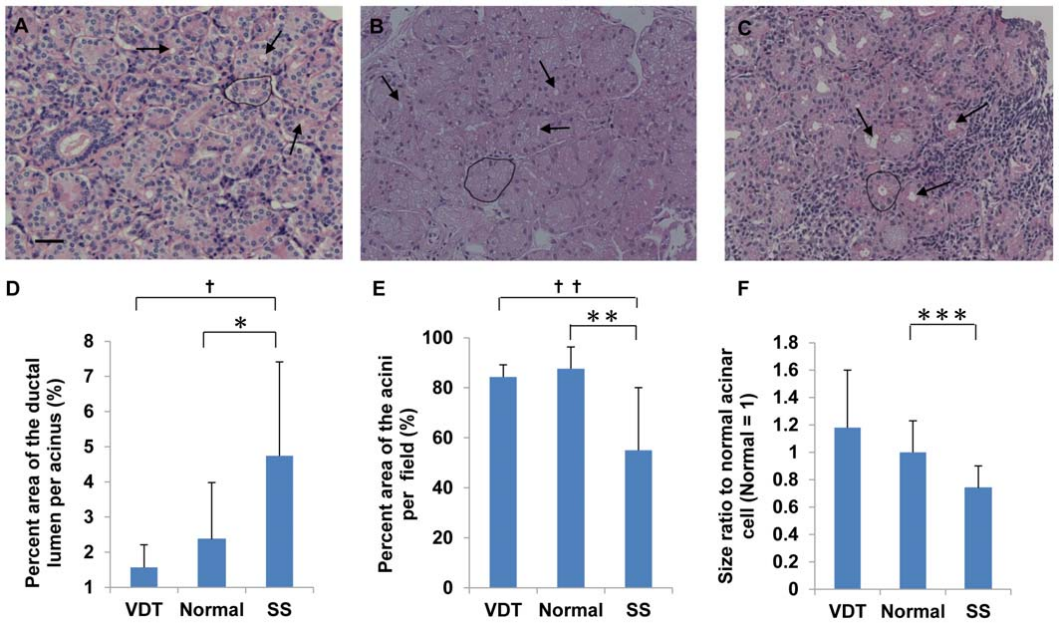
Lacrimal gland histology by H&E staining. (A) H&E staining of lacrimal gland from normal controls (n = 9). (B) H&E staining of lacrimal gland from the VDT group (n = 4). (C) H&E staining of lacrimal gland from the SS group (n = 13). Arrows indicate ductal lumens. Circle indicates one acinus. Scale bars = 50 µm. (D) Percent area of the ductal lumen per acinus. Total number of acini examined/group: 879/VDT group (4 cases), 2241/SS group (13 cases), and 1869/Normal controls (9 cases). *P = 0.03, **^†^** *P* = 0.013 (Mann-Whitney U test). (E) Percent area of the acini per field. Number of fields/group: 12/VDT group (4 cases), 39/SS group (13 cases), and 27/Normal controls (9 cases). **P = 0.001, **^††^** *P* = 0.013 (Mann-Whitney U test). (F) Acinar cell size relative to that of normal acinar cells. Number of cells measured/group: 6668/VDT group (4 cases), 19021/SS group (13 cases), and 17325/Normal controls (9 cases). ***P = 0.01 (Mann-Whitney U test). Original magnification: ×200 (A–C).

In the normal controls, the lacrimal gland structure was maintained despite being obtained post-mortem. The glands consisted of acinar cells, ductal cells, capillaries and connective tissue (Figure 2A) as reported previously [22]–[24]. In the VDT group, the lacrimal gland acini were larger than those in normal controls, and showed ductal obstruction (Figure 2B). Histopathological findings in the lacrimal gland from SS patients showed the destruction of acini with lymphocytic infiltration and ductal dilation (Figure 2C).

The percent area of the ductal lumen per acinus was 1.57±0.64 (n = 4), 2.37±1.62 (n = 9) and 4.74±2.68 (n = 13) in the VDT group, normal controls, and SS group, respectively. This percent in the SS group was significantly higher than in the normal controls (*P* = 0.03) or the VDT group (*P* = 0.013). Although this percent in the VDT group was lower than normal controls, the difference was not significant (Figure 2D).

The percent area of the acini per field was 84.35±4.83 (n = 4), 87.63±8.75 (n = 9), and 55.05±25.06 (n = 13) in the VDT group, normal controls, and SS group, respectively. This percentage in the SS group was significantly lower than in normal controls (*P* = 0.001) or the VDT group (*P* = 0.013). This percentage in the VDT group was the same as in normal controls (Figure 2E).

The size of the individual acinar cells was 1.19 and 0.74 in the VDT and SS groups, respectively, when the size of the normal controls' acinar cells was defined as 1. The size in the SS group was significantly smaller than that of normal control cells (*P* = 0.01). In contrast, in the VDT group, the acinar cells appeared larger than those of normal controls (Figure 2F), although this difference did not reach significance.

These results suggested that the lacrimal gland acini of the VDT group tended to be larger than those of the other groups. In the SS group, the acini of the lacrimal gland were damaged and atrophic. These glands were largely replaced by fibrotic tissue and lymphocytes.

### Electron microscopic findings

**Figure 3:**
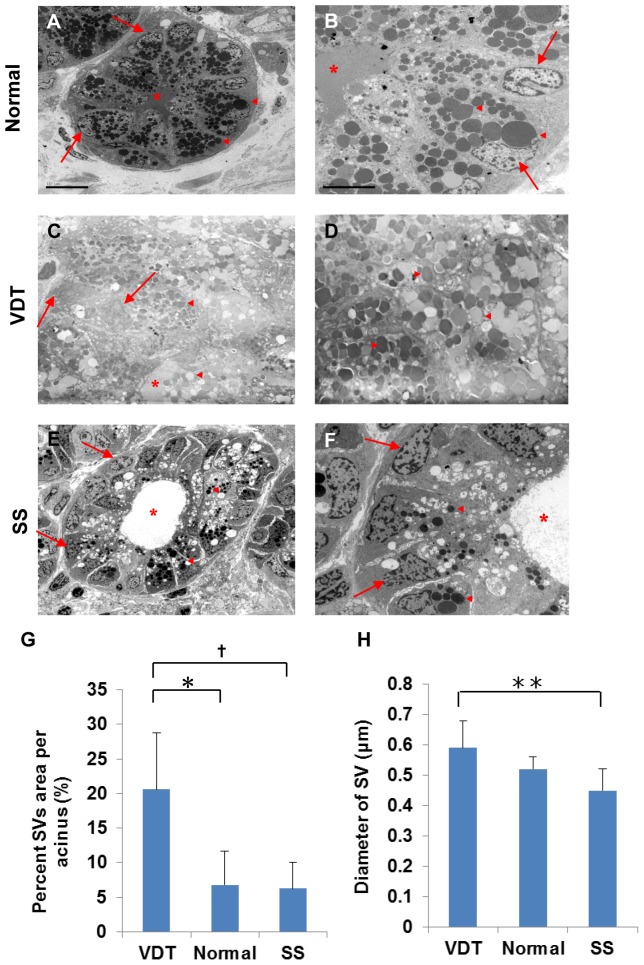
Electron microscopic findings of the lacrimal gland acinus in the three groups. A: SV accumulation in the normal lacrimal gland (n = 4). Scale bars = 10 µm. B: Homogeneous SVs in normal controls. Scale bars = 5 µm. C: Excessive accumulation of SVs in the VDT group (n = 4). D: High magnification view of SVs in the VDT group. E: Only a few SVs in the SS group (n = 12). F: High magnification view of SVs in the SS group. G: Percent SV area per acinus. Total number of acini examined/group: 20/VDT group (4 cases), 58/SS group (12 cases), and 20/Normal controls (4 cases). *P = 0.021, ^†^ *P* = 0.004 (Mann-Whitney U test). H: SV diameter. Total number of vesicles examined/group: 6274/VDT group (4 cases), 11837/SS group (12 cases), and 2875/Normal controls (4 cases). **P = 0.025 (Mann-Whitney U test). Original magnification: ×2000 (A, C, E), ×5000 (B, D, F). Asterisk, Ductal lumen; Arrows, Nuclei; Triangle, SV.

To examine the SV accumulation in detail, we analyzed the ultrastructural morphology of the lacrimal gland using TEM. In normal controls, SVs accumulated homogeneously toward the apical region of the lacrimal gland epithelial cells (Figure 3A, B). In contrast, there was excessive accumulation of SVs in the VDT group (Figure 3C), so that the nuclei were displaced toward the cell periphery by the SVs, which filled the cytoplasm. We noted SVs of both high and low electron density in the VDT group (Figure 3D). There were only a few SVs in the SS group, and dilation of the duct was visible (Figure 3E). The SVs were also smaller in the SS group than in the other groups (Figure 3F), and showed uniform density.

These TEM findings indicated that the VDT group had an unusually large number of SVs in the cytoplasm of the lacrimal gland epithelial cells compared with the other two groups.

### Analysis of the lacrimal gland in TEM images

We next performed a statistical analysis of the percent SV area in the cytoplasm of lacrimal gland epithelial cells from the TEM findings. The percent SV area per acinus was 20.57±8.15 (n = 4), 6.78±4.87 (n = 4) and 6.26±3.81 (n = 12) in the VDT group, normal controls, and SS group, respectively. This percentage in the VDT group was significantly higher than in normal controls (*P* = 0.021) or the SS group (*P* = 0.004). This percentage in the SS group was the same as in normal controls (Figure 3G).

The diameter of the SVs was 0.59±0.09 µm (n = 4), 0.52±0.04 µm (n = 4), and 0.45±0.08 µm (n = 12) in the VDT group, normal controls, and SS group, respectively. The diameter of the SVs in the VDT group also appeared larger than in normal controls (Figure 3H), although the difference was not significant. However, there was a significant difference in the SV diameter between the VDT and the SS groups (*P* = 0.025).

These results revealed that the individual SVs tended to be larger and to occupy more volume in the cytoplasm of the lacrimal gland epithelial cells in the VDT group compared with the other groups. In contrast, the SVs were smaller and occupied less volume in the cytoplasm of lacrimal gland epithelial cells in the SS group.

### VDT duration

We interviewed the VDT group and SS patients regarding their VDT usage for work, watching TV, and playing video games. All the patients in the VDT group performed work using a VDT for more than 5 hours per day. Of the SS patients, 8 did not work with a VDT at all, 6 used it for less than 5 hours per day, and none used it for more than 5 hours per day (Table 3). In addition, the patients in the VDT group had used VDTs for long hours over many years, but the SS patients had not.

**Table 3:** VDT duration.

	VDT (N = cases)	SS (N = cases)
VDT work hour: 0	0	8
<5 hours	0	6
>5 hours	4	0
Unknown	0	1

VDT duration, visual display terminals duration; VDT, VDT related to non-Sjögren's syndrome dry eye; SS, Sjögren's syndrome dry eye.

### Immunohistochemistry-VAMP8

We next examined whether the expression of VAMP8, a molecule involved in the exocrine pathway, was altered in the VDT group. VAMP8 may play a role in regulating exocytosis throughout the exocrine system [25], [26].

**Figure 4:**
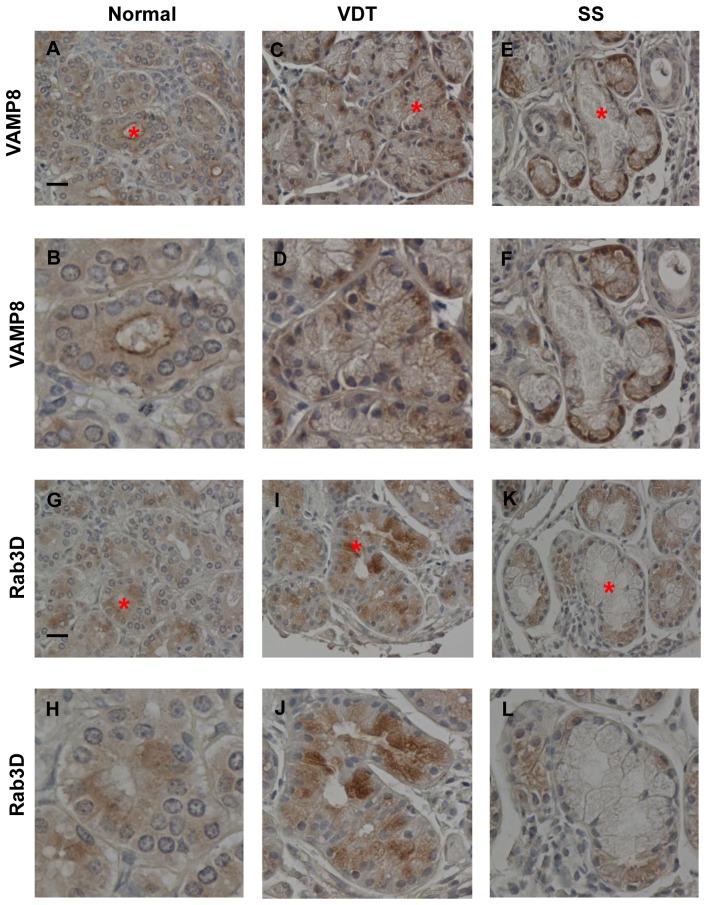
Immunohistochemistry of VAMP8 and Rab3D. (A), (B): VAMP8 expression in normal controls was localized to the apical membrane of the lacrimal gland epithelia (A, B; 87 years old, Female). (C), (D): In the VDT group, strong VAMP8 immunostaining was present in the cytoplasm and basal side of the lacrimal gland epithelial cells but not at the apical membrane (C, D; VDT-case 3). (E), (F): VAMP8 staining in the SS group was seen close to the basal side of the acinar epithelia (E, F; SS-case 8). A, C, E; Original magnification: ×400, B, D, F; Magnified view of the acinus with asterisk in A, C, E. Scale bars = 20 µm. (G), (H): Rab3D expression in normal controls. Rab3D was expressed at the apical side of the cytoplasm in the lacrimal gland epithelia, but not at the apical membrane, where VAMP8 was expressed (G, H; 87 years old, Female). (I), (J): In the VDT group, strong Rab3D immunostaining was observed in the cytoplasm of the lacrimal gland epithelial cells (I, J; VDT-case 3). (K), (L): In the SS group, faint Rab3D expression was observed at the basal side of the acinar epithelia (K, L; SS-case 8). G, I, K; Original magnification: ×400. H, J, L: Magnified view of the acinus with asterisk in G, I, and K. Scale bars = 20 µm.

In normal controls, VAMP8 immunostaining was localized to the apical membrane and cytoplasm of lacrimal gland epithelial cells (Figure 4A, B). In comparison, the distribution of VAMP8 immunostaining in the VDT and SS groups was different (Figure 4C–F). In the VDT group, VAMP8 immunostaining was strong in the cytoplasm and basal side of lacrimal gland epithelial cells, but it was not localized to the apical membrane. The staining in the SS group was detected close to the basal side of the acinar epithelia. Thus, the distribution of VAMP8 was different in each of the three groups (Figure 4 A–F). These results suggested that normal tear secretion in the VDT and SS groups was impaired by disruption of the exocrine pathway, as reflected in the abnormal distribution of VAMP8.

### Immunohistochemistry-Rab3D

Rab3D is primarily localized to mature SVs in lacrimal gland acinar cells [27]. We therefore performed immunohistochemical staining for Rab3D to investigate the maturity of the SVs of the lacrimal gland epithelia in the three groups.

In the normal controls, Rab3D immunostaining was observed at the apical side of the cytoplasm, but not at the apical membrane where VAMP8 was expressed (Figure 4G, H). The Rab3D immunostaining was stronger in the cytoplasm of the lacrimal gland epithelial cells of the VDT group compared to that of normal controls (Figure 4I, J). In the SS group, faint Rab3D staining was detected at the basal side of the acinar epithelia (Figure 4K, L). Thus, the distribution of the Rab3D staining differed among the normal controls, the VDT group, and the SS group (Figure 4 G–L). The strong staining in the VDT group indicated that mature SVs accumulated in the cytoplasm of the lacrimal gland epithelial cells. In contrast, there were few mature SVs in the cytoplasm of the lacrimal gland epithelia in the SS group. These findings indicated that the SVs of the VDT group were generally more mature than those of the SS group.

## Discussion

In the present study, we found that the human lacrimal glands from a VDT-using group exhibited remarkable morphological differences from those of SS patients and normal controls. Specifically, the SVs in the VDT group were significantly increased in the cytoplasm of the lacrimal gland epithelial cells.

Our analysis of H&E stained images showed that the percent area of the ductal lumen per acinus in the VDT group was lower and the size of the individual acinar cells tended to be larger than in the other groups. We ascertained that the increased size of the acini and acinar cells of the VDT group was due to the increased SV accumulation. In addition, our analysis of TEM images showed that the percent SV area per acinus in the VDT group was significantly higher than in normal controls (*P* = 0.021) or the SS group (*P* = 0.004). The TEM findings also revealed excessive SV accumulation in lacrimal gland epithelial cells of the VDT group. Conversely, the analysis of H&E-stained images in the SS group showed acini and acinar cells that were smaller than in the other groups, and TEM showed there were many fewer SVs. The TEM images also indicated that the lacrimal gland structure of the SS group was atrophic, with damaged acini.

In selecting representative TEM photographs in a blinded fashion, we chose cross-sections of lacrimal glands with ducts located in the center of the acinus. Some acinar cells included the nucleus in these cross sections, and some did not. Therefore, we analyzed the percent SV area per acinus for acinar cells with and without a nucleus. We found that both sets of results showed the same trend.

The lacrimal gland specimens we used were very small, to minimize the effects of biopsy on the patients' eye. Sato et al. has reported that lacrimal gland biopsy is a safe and useful procedure. We observed no severe side effects and no difference in the Schirmer's test, fluorescein, and Rose-Bengal staining scores before and after biopsy. Furthermore, lacrimal gland biopsy provides important information for diagnosing Sjögren's syndrome [28].

VAMP8 is a major vesicle SNARE (soluble NSF attachment protein receptor) of SVs. The fusion of an SV with the target compartment is induced by the SNARE complex [25], [26]. Rab3D is a member of the Rab protein family of small Ras-like GTPases that is associated with membrane trafficking that is involved in exocytosis and expressed on mature SVs [27], [29]. In previous reports, VAMP8 and Rab3D immunostaining was detected in the vicinity of the apical membrane and cytoplasm of normal lacrimal gland epithelia. Wang et al. detected VAMP8 immunostaining near the apical membrane in normal mouse lacrimal glands [25]. Evans et al. detected Rab3D immunostaining at the subapical region in normal rabbit lacrimal glands [30]. The immunostaining results for VAMP8 and Rab3D in our normal controls were consistent with previous reports.

In the immunohistochemistry for VAMP8-related exocytosis, the staining distributions in the VDT and SS groups were different from those in normal controls. Instead of being located at the apical membrane as in normal acinar cells [25], [26], the VAMP8 immunostaining in the VDT group was detected in the cytoplasm and at the basal side of the acinar epithelial cells, and that in the SS group was at the basal side of the acinar epithelia. These different distributions indicate that the VDT and SS groups may have disorders of the exocrine pathway that lead to abnormal exocytosis.

Wu et al. reported that Rab3D is present on mature SVs [27]. In the immunohistochemistry for Rab3D in the VDT group, marked staining in the cytoplasm of the lacrimal gland epithelial cells indicated an accumulation of mature SVs. Excessive SVs in the lacrimal gland acini of the VDT group were also revealed by TEM. In this study, our H&E, TEM, and immunohistochemistry results suggested that an unusually large number of SVs accumulated in the cytoplasm of the lacrimal gland epithelial cells in the VDT group. In contrast, the Rab3D staining in the SS group was faint and distributed differently from that of normal controls, suggesting that there were few SVs and/or the SVs were not mature in the SS acinar epithelial cells.

SVs are derived from the trans-Golgi network as large vesicles, and they mature as the intravesicular protein condenses. During this process, the vesicles decrease in size by budding [31]. Even though the SVs of the VDT group were larger than those of normal controls, the difference was not significant. The results of Rab3D immunostaining and SV size in the VDT group indicate that these SVs might have been mature. Conversely, the SVs of the SS group were smaller, and the distribution of Rab3D immunostaining was different from that of normal controls. These findings suggest that the characteristics of the SVs in the SS acinar epithelia are different from those of normal SVs.

VDT-associated dry eye is reported to be induced by the excessive evaporation of tear fluid due to the reduced blinking of the user while gazing at the computer monitor [4]–[8]. However, we recently reported that there are no significant relationships among VDT duration, tear-film break up time (BUT), and DR-1, which represents the precorneal tear stability in VDT users. In contrast, we found a negative relationship between VDT duration and tear secretion in this group [9]. Furthermore, we demonstrated a significant reduction of tear and protein secretion in a rat VDT-user model, with lacrimal gland morphological changes involving an excessive accumulation of SVs, by H&E and TEM analyses. These results suggested that lacrimal gland hypofunction was the critical mechanism for VDT-associated dry eye in this animal model, and that VDT work induced a reduction in tear secretion, rather than excessive evaporation [9].

Another possibility is that reduced stimulation, i.e., blinking, rather than the exocytosis machinery itself leads to dry eye in VDT users. In other words, our data could be explained if the lacrimal gland acinar cells over-compensated for the lack of stimulation by generating excessive SVs. This issue could be clarified by examining the exocytosis capability of the lacrimal gland, which can evaluated by asking subjects to blink after the Schirmer strip has been placed, to measure both the basal and stimulated secretion without anesthesia. We routinely performed this test. In this study, the Schirmer's test values for both types of tearing remained low, suggesting that the accumulation of SVs in the VDT cases was probably not due to overcompensation by the lacrimal gland acinar cells for insufficient stimulation. Therefore, the accumulated SVs in the VDT users may be involved in the reduced tear secretion, but their presence probably does not reflect overcompensation of the lacrimal gland acinar cells due to lack of stimulation.

Based on our findings, we hypothesize a new mechanism for VDT work-related dry eye, in which a tear secretion disorder probably related to a decreased blinking rate leads to an excessive accumulation of SVs. That is, VDT work-related dry eye is likely to be due to a disorder of tear secretion, while tear production appears to be intact. In contrast, the mechanism of SS dry eye is impaired tear production and secretion (Table 4). Intensive, long-term VDT work might trigger lacrimal gland dysfunction.

**Table 4:** Hypothetical mechanism of dry eye in the VDT and SS groups.

	Production	Secretion
Normal	○	○
VDT	○	×
SS	×	×

VDT, VDT work-related to non-Sjögren's syndrome dry eye; SS, Sjögren's syndrome dry eye.

The present study has several limitations. First, there is a possibility of sample bias, because very small human specimens were excised to avoid affecting the patients' dry-eye status. Second, we could not carry out all the histological examinations for every case, because the sample volumes were limited. Third, there are unresolved age- and sex-dependent effects on histological changes, innervation, and secretory responses [32]. However, we could not adjust our study population for age, gender, or number, or perform time-course experiments, for ethical reasons and because of the limited number and small size of our human samples.

We are unaware of any previous report on human dry eye showing a correlation with SVs in these three groups. We believe that this study provides new insight for the mechanism of dry eye and lays the foundation for the development of new treatments. Further study will be required to better understand the mechanisms of dry eye.
